# The Potential Biomarkers and Immunological Effects of Tumor-Derived Exosomes in Lung Cancer

**DOI:** 10.3389/fimmu.2018.00819

**Published:** 2018-04-18

**Authors:** Shamila D. Alipoor, Esmaeil Mortaz, Mohammad Varahram, Mehrnaz Movassaghi, Aletta D. Kraneveld, Johan Garssen, Ian M. Adcock

**Affiliations:** ^1^Molecular Medicine Department, Institute of Medical Biotechnology, National Institute of Genetic Engineering and Biotechnology (NIGEB), Tehran, Iran; ^2^Clinical Tuberculosis and Epidemiology Research Center, National Research Institute of Tuberculosis and Lung Diseases (NRITLD), Shahid Beheshti University of Medical Sciences, Tehran, Iran; ^3^Department of Immunology, Faculty of Medicine, Shahid Beheshti University of Medical Sciences, Tehran, Iran; ^4^Mycobacteriology Research Center, National Research Institute of Tuberculosis and Lung Disease (NRITLD), Shahid Beheshti University of Medical Sciences, Tehran, Iran; ^5^Division of Pharmacology, Faculty of Science, Utrecht Institute for Pharmaceutical Sciences, Utrecht University, Utrecht, Netherlands; ^6^Faculty of Veterinary Medicine, Institute for Risk Assessment Sciences, Utrecht University, Utrecht, Netherlands; ^7^Nutricia Research Centre for Specialized Nutrition, Utrecht, Netherlands; ^8^Airways Disease Section, Imperial College London, National Heart & Lung Institute, London, United Kingdom; ^9^Priority Research Centre for Healthy Lungs, Hunter Medical Research Institute, The University of Newcastle, Newcastle, NSW, Australia

**Keywords:** tumor-derived exosome, lung tumor, exosomes, microRNA, NSCLC

## Abstract

Lung cancer remains the leading cause of cancer-related deaths worldwide. Despite considerable achievements in lung cancer diagnosis and treatment, the global control of the disease remains problematic. In this respect, greater understanding of the disease pathology is crucially needed for earlier diagnosis and more successful treatment to be achieved. Exosomes are nano-sized particles secreted from most cells, which allow cross talk between cells and their surrounding environment *via* transferring their cargo. Tumor cells, just like normal cells, also secrete exosomes that are termed Tumor-Derived Exosome or tumor-derived exosome (TEX). TEXs have gained attention for their immuno-modulatory activities, which strongly affect the tumor microenvironment and antitumor immune responses. The immunological activity of TEX influences both the innate and adaptive immune systems including natural killer cell activity and regulatory T-cell maturation as well as numerous anti-inflammatory responses. In the context of lung cancer, TEXs have been studied in order to better understand the mechanisms underlying tumor metastasis and progression. As such, TEX has the potential to act both as a biomarker for lung cancer diagnosis as well as the response to therapy.

## Introduction

Lung cancer is one of the most common cancers and the leading cause of cancer-related death worldwide. The two histological subtypes of lung cancers are non-small cell lung cancer (NSCLC) that encompasses >80% of lung cancers, including adenocarcinoma, squamous-cell carcinoma, and large-cell carcinoma and small cell lung cancer, which accounts for the remaining 20% of cases (1).

Exosomes are small vesicles (30–100 nm in size) that originate from most cells and are released into biological fluids, such as saliva, plasma, urine, and breast milk. Exosomes enable cell-to-cell communication by transferring their contents including RNA (mRNA and non-coding RNA), DNA (mtDNA, ssDNA and dsDNA), proteins, and lipids (2). This communication influences physiological process of the recipient cell and may be involved in pathological conditions such as cancer (3).

**Figure 1:**
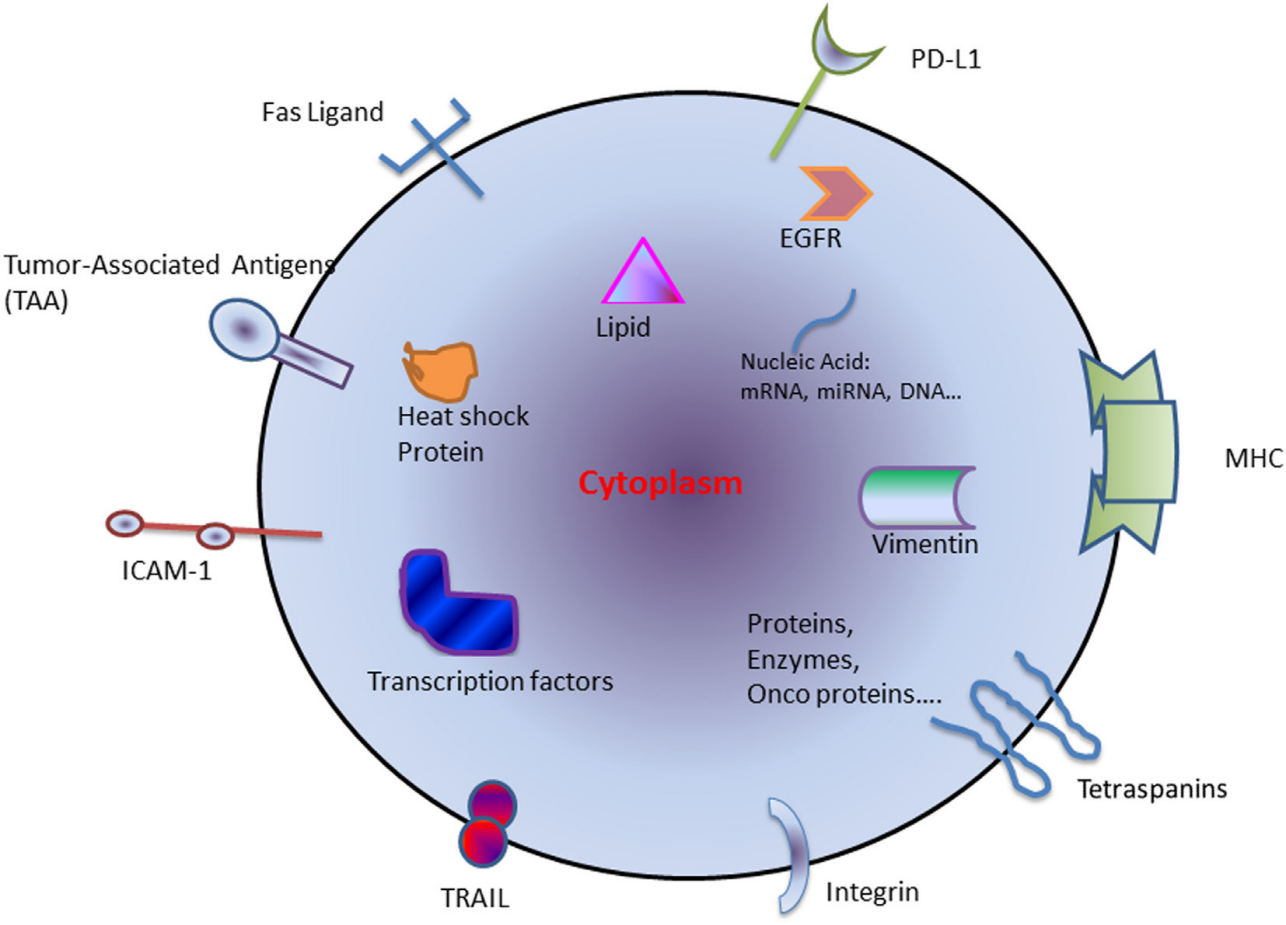
Schematic diagram of components generally found within tumor-derived exosome. Abbreviations: HSP, heat-shock protein; miRNA, microRNA; TAA, tumor-associated antigen; TRAIL, tumor-necrosis-factor-related apoptosis-inducing ligand; ICAM-1, intercellular Adhesion Molecule 1; PD-L1, programmed death-ligand 1; MHC, major histocompatibility complex; EGFR, epidermal growth factor receptor.

The exosomes derived from tumor cells are called tumor-derived exosomes (TEX) (4) (Figure 1). TEXs are the main mechanism of intracellular communication between tumor and host cells and enable cancer cells to modulate their surroundings to favor an optimal microenvironment for tumor initiation and progression. TEX contains a variety of different immuno-stimulatory and immuno-inhibitory factors that support the cellular reprogramming of the recipient cells. For example, exosomes are involved in promoting cancer growth by transfer of oncoproteins such as K-RAS and MET or oncogenic miRNAs to otherwise healthy cells (5). TEX may also drive metastasis by creating a pre-metastatic niche and directing the disseminated tumor cells to future metastatic sites (6, 7). Interestingly, this does not occur randomly and is regulated by integrin expression on the TEX (8). In contrast, TEX can also induce an antitumor immune response by modulating killer cell lectin-like receptor K1 (KLRK1 or NKG2D) expression on natural killer cells (NKs) and thereby affecting their function (9, 10).

In this article, we review the immunological effects and function of TEX in cancers with an emphasis on lung cancers development and metastasis. Since NSCLC encompasses >80% of lung cancers, so we summarized recent research preferentially for this type of lung cancer. In addition, we evaluate the potential of these exosomes to act as a diagnostic biomarker in lung cancer.

## The Role of TEX in the Lung Tumor Microenvironment

**Figure 2:**
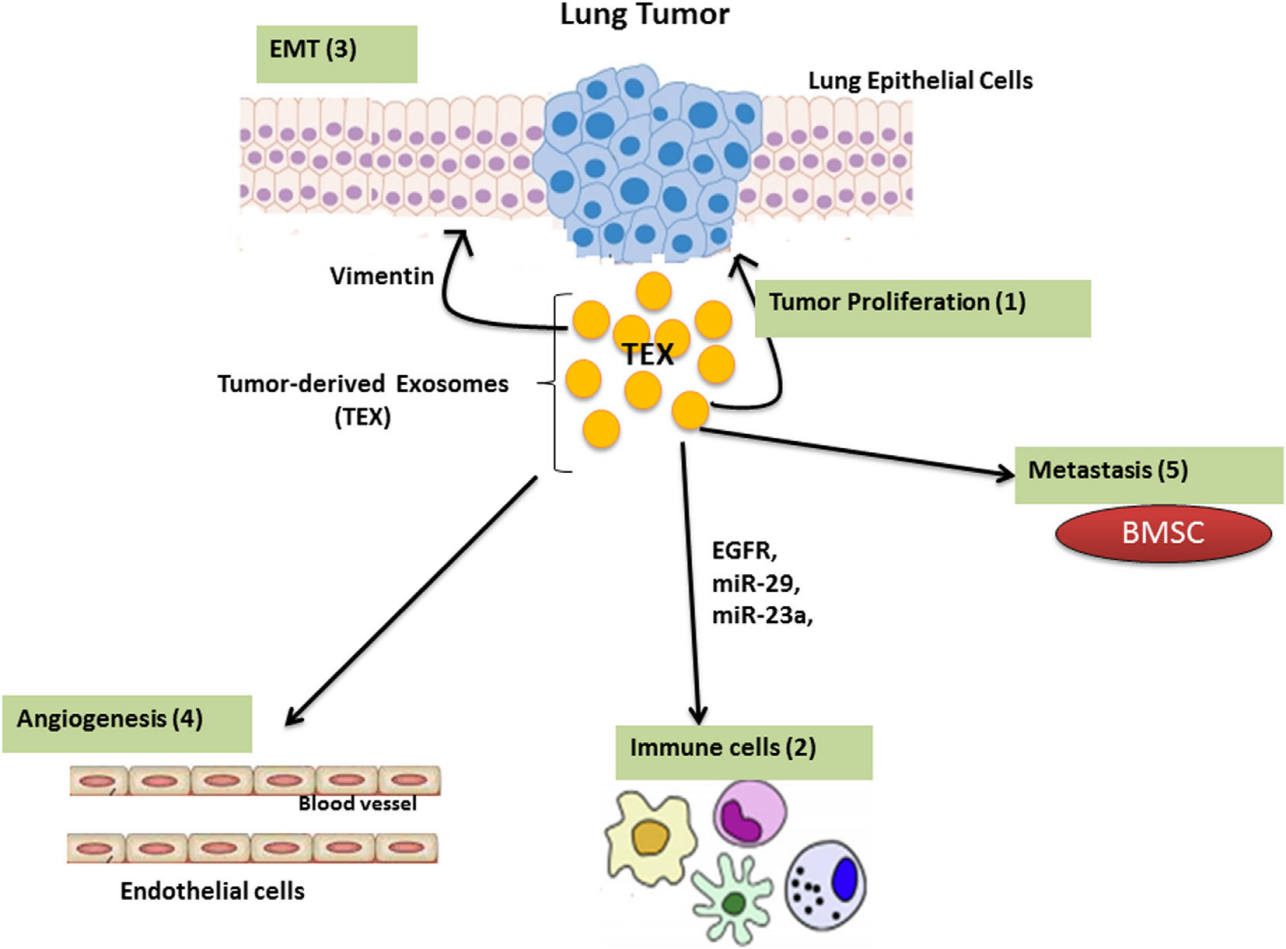
The function of Lung tumor-derived exosome (TEX). TEX impact upon the tumor microenvironment by enhancing tumor cell growth and progression (1); modulating immune responses (2); regulating epithelial–mesenchymal transition (EMT) (3); angiogenesis (4); as well as inducing metastatic behavior in bone marrow progenitors (5).

The tumor microenvironment consists of different components with various properties based on the tumor’s origin. The most abundant components in the tumor microenvironment are: carcinoma cells, immune cells, extracellular matrix (ECM), and stromal tissues (11, 12). The molecular and cellular nature of the tumor microenvironment determines malignancy by modulating local immune responses (13). TEX contain stimulatory and inhibitory components that, when delivered to the recipient cells, enable crosstalk between tumor cells and its surrounding environment. TEXs are involved in modulating the immune response, regulating epithelial–mesenchymal transition (EMT) and cancer-associated fibroblast function as well as playing a key role in angiogenesis (Figure 2).

### Tumor-Derived Exosomes and the Immune Response

**Figure 3:**
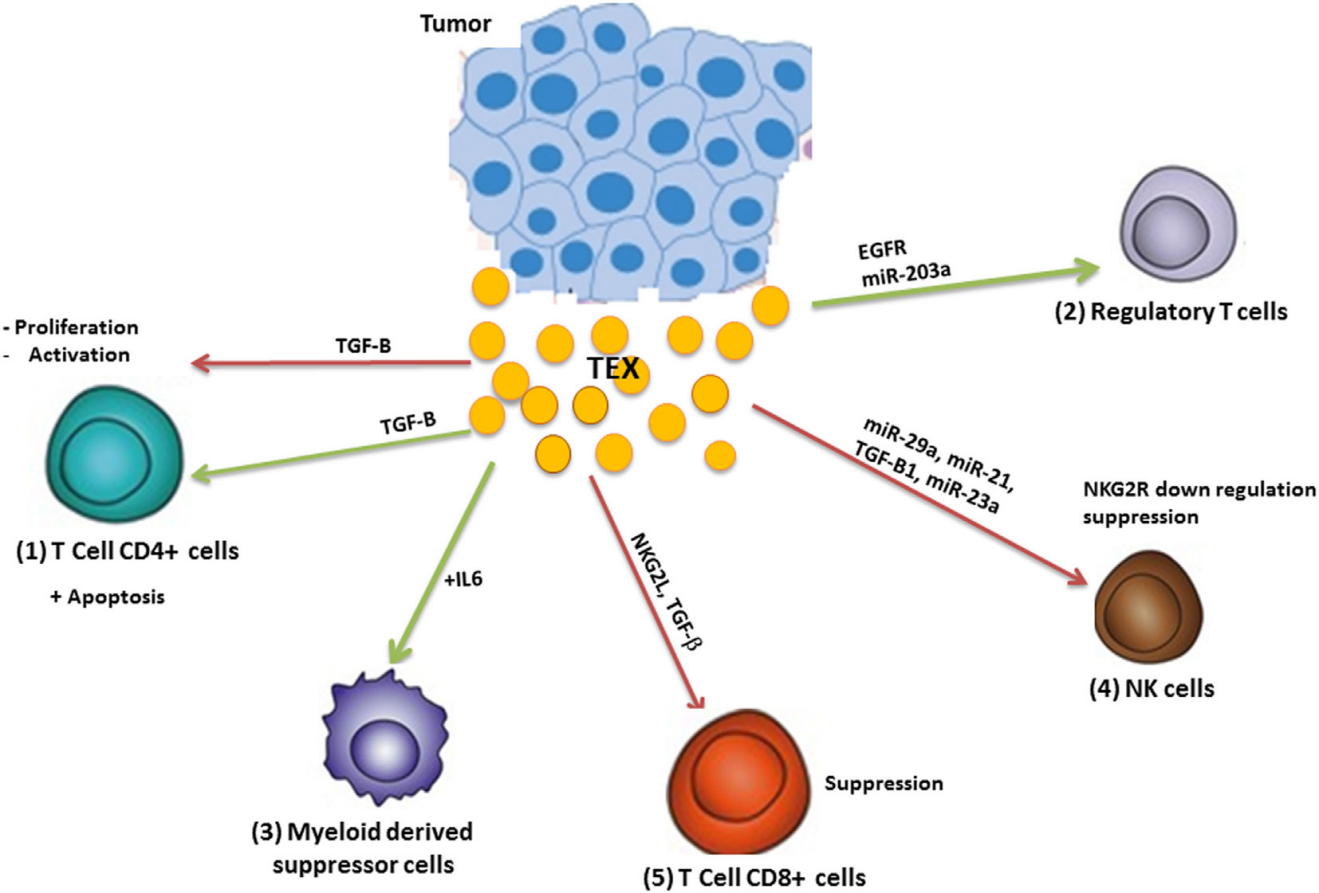
Modulation of the immune system by tumor-derived exosome (TEX). TEX modulate antitumor immune responses by (1) inhibiting T-cell activation and proliferation as well as apoptosis induction; (2) TEXs induce regulatory T-cells and (3) myeloid-derived suppressor cells and inhibit the function of natural killer (NK) and CD8^+^ T-cells (4). Green arrows: induction or stimulation; red arrows: inhibition.

The immune system has a significant impact on cancer outcomes (14). The immune system acts like a double-edged sword in cancer by destroying cancer cells and suppressing tumor growth as well as supporting the chronic inflammation and suppressing antitumor immunity which leads to tumor progression (15) (Figure 3).

#### Natural Killer Cells

Lung cancer cell-associated TEX contain miR-21 and -29a which can both bind to intracellular toll-like receptors (TLRs) on immune cells including NKs, and thereby trigger a pro-metastatic inflammatory response due to activation of NF-κB eventually resulting in metastasis and tumor growth (16).

In NKs, the C-type lectin-like receptor NKG2D serves as an activating receptor to trigger cytotoxicity toward cancerous cells that express its ligand (17). TEX originating from hypoxic tumor cells inhibit NK function by delivering transforming growth factor (TGF)-β1 to NKs and subsequently reducing NKG2D expression (18). In a mouse model, TEX reduced the percentage of NKs found within the lungs (19). The downregulation of cell-surface receptors particularly that of NKG2D, may account for the reduced activity of NKs seen in lung cancer patients (20). miR-23a derived from TEX may directly target CD107a, a molecule that protects NKs from granule-mediated degradation (21).

Tumor-derived exosome can also downregulate NKG2D expression on NKs by shedding the NKG2D ligand on tumor cells. This will result in receptor desensitization and internalization and lower activity of NKs (22–25). TEXs may also attenuate NK activity *via* other mechanisms including the down-modulation of interleukin (IL)-2-mediated pathways (26), suppressing perforin or cyclin D3 production (19) and janus kinase (Jak)3 activation resulting in a failure of NK-mediated cytolysis (19).

#### Dendritic Cells (DC) and Myeloid-Derived Suppressor Cells (MDSCs)

It is well-known that tumor microenvironment educate DCs to promote tumorigenicity. TEXs have important roles in this context by shuttling signaling molecules and tumor antigens and developing cell-to-cell communication (27).

Approximately 80% of the exosomes isolated from lung cancer biopsies contain epidermal growth factor receptor (EGFR) which has the potential to induce tolerogenic DC and regulatory T-cells, ultimately leading to the suppression of tumor antigen-specific CD8^+^ cells (28). In pancreatic cancer, TEX contain mir-203a, that decrease the expression of TLR4 on DCs and subsequently leads to a reduced production of downstream cytokines including tumor necrosis factor (TNF)-α and IL-12 (28, 29) which result in dysfunction of DC and cellular immunity (29). TEXs can also prevent DC maturation and function. In a murine delayed-type hypersensitivity (DTH) model, administration of TEXs loaded with ovalbumin result in suppression of DTH responses by inhibiting DC maturation *via* TGF-β1. This result highlights the roles of TEXs in the promoting tumor antigen-specific immunosuppression, possibly by modulating the function of DCs (30). In melanoma and colon cancer, TEXs promote the differentiation of CD14^+^ monocytes to MDSCs rather than to DCs (31). MDSCs are an immature population of myeloid cells identified in humans and mice that expand in cancer and have strong immunosuppressive effects on the antitumor T-cell response (32). TEX interaction with monocytes, results in a monocyte phenotype that is characterized by a failure to upregulate co-stimulatory molecules (29, 33) and decreased human leukocyte antigen-DR expression (34, 35) with unchanged CD14 surface expression (35). Collectively, TEXs alter monocyte differentiation to DCs and promote the maintenance of an immature monocyte status. These cells spontaneously secrete immune inhibitory cytokines such as TGF-β and prostaglandin E2 which inhibit T-cell proliferation and antitumor functions (31).

However, the overall effect *in vivo* is likely to be complex. Intravenous injection of TEXs into mice resulted in the accumulation of MDSCs and a marked increase in the production of inflammatory mediators, including IL-6 and vascular endothelial growth factor (VEGF) (36). On the other hand, the accumulation of MDSCs increased the production of immunosuppressive factors, such as nitric oxide and reactive oxygen species, which cause T-cell apoptosis (31). Both of these processes lead to tumor progression.

The presence of heat-shock protein 72 (HSP72) on the surface of TEXs, could trigger the activation of STAT3 and autocrine IL-6 production in MDSCs in a TLR2/MyD88-dependent manner which promotes the suppressive activity of MDSCs (37–39). Treatment of mice with TEX significantly increased tumor metastasis along with the recruitment of MDSCs into the lung. These effects were mediated by MyD88 which is a cytoplasmic adaptor molecule needed for the integration and transduction of TLR signaling (24).

#### Tumor-Associated Macrophages (TAMs)

Tumor-associated macrophages are the major modulators of the tumor microenvironment that regulate angiogenesis, invasion, metastasis, as well as immunosuppression in tumor stroma (40). During tumor progression, circulating monocytes and other inflammatory lymphocytes are recruited into tumor tissue and alter tumor microenvironment. Monocytes are the precursors of TAMs that can get a continuous survival subsist in the inflammatory tumor microenvironment and generate TAMs (41). TEXs have a pivotal role in monocyte survival and in TAM generation within the tumor inflammatory niche. TEXs trigger the mitogen-activated protein kinase (MAPK) pathway in monocytes through delivery of functional receptor tyrosine kinase, which in turn leads to inhibition of apoptosis-related caspases (42).

Hsp72 and palmitoylated proteins on the TEX surface also modulate TLR signaling and the function of TAMs, which have a critical role in reinforcing tumor metastasis and invasion. Thus, TEX, acting through TLR2 and triggering the NF-κB signal pathway can promote the secretion of pro-inflammatory cytokines by macrophages (43). The induction of breast cancer invasion and metastasis by TAMs requires the upregulation of Wnt5α in macrophages that leads, in turn, to the activation of β-Catenin-independent Wnt signaling in tumor cells. TEX mediate the crosstalk between tumor cells and TAMs and TAM-derived exosomes have a reciprocal supportive role in providing factors that activate β-Catenin-independent Wnt signaling in the breast cancer cells (44). This reciprocal interaction of TEX and TAM-derived exosomes may maintain TAM survival within the inflammatory niche (45). In lung cancers, interaction between TAMs and tumor cells results in tumor progression *via* STAT3 activation and TAM-derived IL-6 (46). This may simply be a result of the nature of exosomes in cell–cell communication and in shuttling signaling molecules.

Exosome released from TAMs also can be important on cancer progression. Comprehensive proteomic analysis showed TAM-derived exosomes have different proteomic signature and higher proteolytic activity (47). In epithelial ovarian cancer (EOC), TAMs derived exosomes inhibit the migration endothelial cells (ECs) by targeting the miR-146b-5p/TRAF6/NF-κB/MMP2 pathway. On the other hand, EOC-derived exosomes reverse this effect on ECs by transferring long non-coding RNAs (48). Exosomes derived from TAMs also are involved in induction of cisplatin resistance in gastric cancer by transferring miR-21 (49). Overall, the existence of different mechanisms for several cancer cells exosome types in tumor microenvironment enforces the role of exosomes as the major player in cancer progression.

#### T-Regulatory (Treg) and B-Regulatory (Breg) Cells

In contrast to immune cells such as NKs, B-cells, and monocytes that internalize TEX, TEX induce a Ca^2+^ influx in T-cells in the absence of exosome internalization. Plasma TEX from cancer patients cause a strong and sustained increase in inosine production in Treg cells which suggests a functional consequence of TEX signaling on these recipient cells (50). TEXs also enhance Treg and Breg proliferation via TGF-β and IL-10-dependent mechanisms and thereby increase their resistance to apoptosis (20, 31).

The level and suppressor activity of Tregs are higher in the peripheral blood of patients with cancer compared to healthy subjects (51). This may reflect the ability of TEXs to stimulate Treg expansion, increase their resistance to apoptosis and enhance their suppressor activity (52, 53).

Tumor-derived exosomes induce the conversion of CD4^+^CD25^neg^ T cells into CD4^+^CD25^high^FOXP3^+^ Treg cells. On the other hand, incubation of Treg with TEXs, increased the expression of FasL, IL-10, TGF-β1, cytotoxic T-lymphocyte associated protein 4 (CTLA-4), granzyme B and perforin as well as Smad2/3 and STAT3 phosphorylation in Tregs (52).

Tumor-derived exosomes stimulate the expression of CD39 and adenosine production in Treg *via* modulation of related genes in the adenosine pathway (4). Treg produce adenosine *via* ATP hydrolysis by both CD39 (ATP-hydrolase) and CD73 (5′-nucleotidase) on their surface. Adenosine is an immunosuppressive factor that suppress T cell function by binding to its receptors A1, A2A, A2B, and A3. TEX contain surface CD39 and CD73, directly deliver membrane-tethered CD73 to CD39^+^cells and negatively modulate T cells function by production of extracellular adenosine and thus decrease the local immunity (54). These TEX-mediated mechanisms are important in regulating tolerance of tumor and can promote tumor invasion in cancers. TEXs also induce loss of CD69 on the surface of conventional CD4^+^T (Tconv), which leads to their functional decline (4).

Regulatory B cells (Breg) are a subset of B cells with immunosuppressive properties that mediate immunological tolerance. Breg produce molecules, such as IL-10, IL-35, TGF-β, programed death-ligand 1 (PD-L1), and IL-21, and induce the production of Treg and thereby prevent immunopathologic events by inhibition of pro-inflammatory lymphocytes (55). Elevated levels of regulatory Bregs are reported in PBMCs of invasive carcinoma of breast cancer patients (56).

It was shown that exosomes released from mycoplasma-infected tumor cells preferentially activate IL-10-producing B cells which in turn inhibit T cell activity (57). Exosomes released from the esophageal cancer cells also induce Breg production. These microvesicles carried LAMP1 and matrix metalloproteinase (MMP)9 and induce differentiation naive B cells into TGF-β-producing regulatory B cells which subsequently suppress CD8^+^ T-cell activities (58). These information highlight the importance of TEXs in tumor immunity by the mechanisms involved in modulation of Tregs and Bregs.

## The Role of TEX in EMT in Lung Cancer

Epithelial–mesenchymal transition is a process by which epithelial cells acquire mesenchymal cell properties. In this process, the epithelial cells lose their cell polarity and adhesion properties and gain a motile trait, which gives them an invasive character (59). This enables the epithelial cell to migrate to distant sites allowing metastasis and tumor progression (60). EMT is also important in providing the stemness characteristics of cancer cells by supporting the correct microenvironment (60). The importance of EMT in cancer, particularly lung cancer, has been highlighted (60–62). TEX isolated from the serum of late stage lung cancer patients, like highly metastatic lung cancer cells, contain high levels of vimentin and the TEX can induce EMT in recipient human bronchial epithelial cells (63). Vimentin, a member of the type III intermediate filament protein family, is normally expressed in mesenchyaml cells and is widely used as a marker for EMT (64). The association of vimentin expression with increased metastasis and invasion ability has been reported for many cancers including lung (63, 65, 66), prostate (67, 68), and gastric cancers (69). In lung cancer, vimentin changes cancer cell adhesion by regulating the VAV2–Rac1 pathway and modifying focal adhesion kinase activity (65). EMT induction in epithelial adenocarcinoma A549 cells by TGF-β leads to the production of exosomes with a different cargo (70). Exosomes from mesenchymal-like A549 cells contain high levels of β-catenin, vimentin, and E-cadherin, as well as miR-23a in comparison to those from epithelial-like A549 cells. miR-23a mediates TGF-β-induced A549 cell EMT by targeting E-cadherin in a smad-dependent manner (71). Interestingly, autologous treatment of A549 cells with these exosomes induced overexpression of β-catenin indicating the potential for autocrine signaling by TEX (71).

## Angiogenesis Enhancement by TEX in Lung Cancer

Angiogenesis or the formation of a vasculature network is essential for tumor growth and metastasis. This process is regulated by different mechanisms and angiogenic factors, including VEGF, TGF-β, and fibroblast growth factor. Exosomes have a crucial role in vascular tube formation and the observed effect is dependent upon the site of exosome origin (72). Hypoxia is a hallmark of the tumor microenvironment and is reported to lead to an increase in TEX production by tumor cells and a change in their content. The change in TEX cargo under the hypoxic conditions enables them to alleviate the stress conditions in the tumor microenvironment by induction of angiogenesis (73, 74) In CL1-5 lung adenocarcinoma cells, TEX production and the level of TEX miR-23a was enhanced during hypoxia-induced angiogenesis. Uptake of TEX-associated miR-23a by ECs enabled targeting of prolyl hydroxylase 1 and 2 (PHD1 and 2) leading to the accumulation of hypoxia-inducible factor (HIF)-1α and the enhancement of angiogenesis (75).

Tissue inhibitor of metalloproteinases (TIMP)-1 is a factor that strongly supports lung cancer progression (76–79) and its expression is elevated in all stages and types of lung cancer particularly in adenocarcinoma (73). Overexpression of TIMP-1 induces the expression of the tumorigenic miR-210 in lung adenocarcinoma cells and within their derived exosomes under the control of the PI3K/Akt/HIF-1 pathway. In turn, TEX released from these cells downregulate Ephrin A3 in ECs and promote angiogenesis (73). The expression of TEX miR-210 in the serum of lung cancer patients is increased compared to non-cancerous control subjects (80, 81).

Tumor-derived exosome from lung tumor cells contain EGFR and uptake of TEX by ECs can trigger EGFR-dependent responses which are accompanied by the autocrine activation of VEGF receptor 2 (VEGFR-2) and elevated VEGF expression promoting angiogenesis (82). Furthermore, administration of TEX from a lung cancer patient into a rat critical limb ischemia model markedly augmented the expression of VEGFR-2, increased angiogenesis and improved blood flow (83). Together, these observations indicate the important role of TEX in the upregulation of tumor angiogenesis.

## Lung Cancer Metastasis and TEX

The primary step required for metastasis is the formation of a pre-metastatic niche: a supportive microenvironment in a secondary organ that enables its colonization by circulating tumor cells (CTCs) (84). The site of metastasis is not random but is selected following modification by tumor cells before the initiation of metastasis (85). In contrast, the metastatic niche is initiated and formed upon CTC arrival (86). The formation of the pre-metastatic niche is initiated through a variety of mechanisms that promote a sequence of events that begins with vascular leakage. In the lung cancer vascular permeability increases upon upregulation of angiopoietin 2 (Angpt2), MMP3, and MMP10 in the pre-metastatic stage (87). Texosomes can increase vascular permeability at lung pre-metastatic sites by reprogramming bone marrow (BM) progenitors within the niche toward a provascular phenotype *via* the MET receptors. Finally; vascular leakiness facilitate extravasation and attraction of CTCs to the pre-metastatic site (88).

It is now evident that TEX has important roles as mediators in the formation of pre-metastatic niches and the resultant metastasis (89, 90). The role of exosomes in lung metastasis was first demonstrated by Janowska-Wieczorek et al. in 2005 (91). The authors showed that microvesicles derived from activated platelets (PMV) induce tumor progression, metastasis, and angiogenesis in lung cancer. Intravenous injection of pmv-covered Lewis Lung Cancer cell line (LLC) enhanced lung metastasis. These vesicles transferred the integrin α2β (CD41) to lung cancer cell lines and subsequently promote proliferation and tumor progression in mice (91). In addition, renal cancer stem cells trigger an angiogenetic switch and tumor progression and play important role in lung pre-metastatic niche formation (92).

The small RNA content of lung TEX promotes the formation of a pre-metastatic niche by selectively targeting and activating TLR3 in lung epithelial cells. This results in enhanced chemokine secretion and subsequent neutrophil recruitment to the lung which together promotes pre-metastatic niche formation (93).

Melanoma-derived TEXs are important in the primary tumor formation and lung metastasis. Intravenous injection of labeled TEX into a naïve mouse, demonstrated lung residency within 24 h associated with an increased permeability of lung ECs at the TEX-induced pre-metastatic niche. Upregulation of pre-metastatic niche effector molecules such as S100A8 and S100A9 as well as the vascular permeability factor TNF-α was also observed at the site of TEX injection (94). In addition, TEX administration caused an upregulation of inflammatory and ECM-related genes (95). Importantly, TEX obtained from highly metastatic melanomas had a greater burden on the lung compared to those obtained from poorly metastatic melanomas. It is proposed that these melanoma TEXs promote pre-metastatic niche formation and tumor growth by overexpressing the oncogene MET within BM-derived DCs to obtain a pro-vasculogenic phenotype. In support of this, TEX re-program BM progenitors to increase the pro-angiogenic c-Kit ^+^Tie2^+^ cell population in the lung pre-metastatic niche. In addition, TEX could also transfer the oncogene MET from melanoma cells to BM progenitor cells and thereby promote metastasis (95).

Exosome target cells selection is determined by their surface adhesion molecules such as integrin. Specific integrin profiles on the surface of tumor-derived exosomes direct them to a specific organ, so driving metastatic organotropism (8). For example α6α4 integrin heterodimer target exosomes to lung PMNs. Lung fibroblasts with upregulated s100 genes, are the main cells that uptake these exosomes and drive PMN formation (8). Exosomes derived from the 4175-LuT breast cancer cells have α6β4 and α6β1 integrins on their surface and localize in regions of the lung which are rich in laminin and promote lung metastasis (8).

Bone is the common metastatic site for NSCLC which result in osteolytic lesions (96). In NSCLC, EGFR is upregulated (97) and amphiregulin (AREG), an EGFR ligand, is packaged in exosomes derived from lung cancer cells (98, 99). NSCLC-exosomes containing AREG, active the EGFR pathway in pre-osteoclasts which leads to an increase in the expression of RANKL and proteolytic enzymes in turn, triggering a vicious cycle driving osteolytic bone metastasis (100). Conversely, extracellular vesicles released from the highly metastatic bone tumors are localized preferentially to lung and can derive metastatic behavior (101).

The lung is the common target for many metastatic primary tumors (102–104) but the precise molecular mechanism behind this tissue-specific metastasis is not completely understood. It is illustrated that lung microenvironment promote the formation of PMN and possibly TEXs play the key roles in this process. Further studies will more clear that the mechanism of specific exosomes effect on tumor microenvironment and promoting lung invasion along with that of other organs.

## The Role of TEX as a Biomarker and Their Therapeutic Implications in Lung Cancer

Despite considerable achievement in both diagnostics and treatment, the global control of lung cancer remains problematic (105). This lack of success is attributed to a failure of early disease detection due to an absence of reliable biomarkers (106). Biomarkers serve as indicators of a particular physiological or biological state in the body and are important in medicine to distinguish a normal or pathogenic condition or/and a response to a therapy (107). In the context of cancer, biomarkers can be prognostic and predictive markers for the risk of progression, recurrence or the effectiveness of a therapeutic intervention (108). Due to their contents reflecting abnormalities in the parent cells and their stability in most biological fluids, exosomes have potential to serve as a promising “liquid biopsy” biomarkers of lung cancer (106, 109). Importantly, in comparison to tissue biopsy that requires surgery, exosomes-based biomarkers would provide a non-invasive diagnostic approach (106).

Exosomal markers such as proteins and non-coding RNAs have been measured in lung cancer. The analysis of 49 proteins attached to the membrane of plasma exosomes of 276 NSCLC patients indicated that some of these proteins including NY-ESO-1 had a significant correlation with survival (110). Microarray-based analysis of serum exosomal miRNAs in NSCLC patients showed a significant upregulation of miR-21 and miR-4257 in patients with a recurrence of the disease (111). In addition, TEX from NSCLC patients has increased EGFR presence (112). These EGFR-contained TEX activate MAPK and Akt/protein kinase B pathways in recipient ECs resulting in VEGF overexpression and increased tumor vascularity (113).

The exosomal expression of two miRNAs associated with Tumor suppression, namely miR-51 and miR-373, was decreased in lung cancer patients and this reduction was associated with poor prognosis (114). Other exosomal miRNAs have been reported as markers of therapeutic response in lung cancer. For example, miR-208a and miR-1246 bind to p21 and DR5 mRNAs, respectively, to promote tumor growth and resistance to radiotherapy (113).

TEX-based markers may provide higher sensitivity and specificity in cancer diagnostics over conventional biopsy methods which require surgery. However, the lack of standardized methods for isolating pure exosome populations and the heterogeneity in cancer-derived exosomes present problems (108). Despite these concerns, there is much interest in TEX-based miRNAs in lung cancer with efforts made to combine purified TEX with next generation sequencing or proteomic analysis to achieve greater insight into TEX-based lung cancer diagnosis. Exosomal miRNA studies report miR-378a, miR-379, miR-139-5p and miR-200b-5p (115), miR-21 (80, 111, 116), miR-155 (116), miR-23b, miR-10b-5p (80), and miR-4257 (111) that vary in the expression level in lung cancer patient in compare to healthy subjects.

However, recently a method of the using Surface-Enhanced Raman Spectroscopy (SERS) combined with principle component analysis (PCA) was suggested for classification of exosomes based on their specific surface pattern of protein and lipids. Lipid and membrane proteins results in a specific Raman spectra; thus, the tumor-derived exosomes and normal cell-derived exosomes vary in their Raman spectral patterns. In this study, lung cancer cell-derived exosomes were differentiated from those from normal cells by 95.3% sensitivity and 97.3% specificity (117). Current challenges in exosome biology using conventional methods include the need for large amounts of highly concentrated sample and the presence of heterogeneity in cancer-derived exosomes. This approach combining SERS with PCA analysis may be good choice to be translated in clinical practice (117).

In another study by Ueda et al., the mass spectrometric quantification of 1,369 exosomal proteins in 46 serum samples of patient with advanced stage of NSCLC demonstrated CD91 as a lung adenocarcinoma specific antigen on exosomes surface (118). Jakobsen et al. also identified a profile of serum exosomal protein in NSCLC patient with advanced stage of disease. In this study, the authors performed a multivariate extracellular vesicle array (EV Array) approach to phenotype plasma exosomes and the results identified a panel of 30 exosomal surface protein marker including CD91, CD317, and EGFR which could distinguish 75% of the patients correctly. This result suggests that EV Array analysis as a potential complementary method in diagnosing NSCLC (119). Exosomal proteins were also investigated in body fluids to survey exosomal biomarkers. Proteomic mass spectrometry showed that leucine rich alpha-2-glycoprotein 1 (LRG1) was highly expressed in urinary exosomes and also in cancer tissues from NSCLC patient in compare to healthy subjects (120). CD171 and CD151 and tetra-spanin 8 was also suggested as potential diagnosis biomarker for NSCLC (121).

Besides diagnostic approaches, exosomes have been considered as suitable vehicles for drug and nucleic acid delivery to target organs. It was demonstrated that bEND.3 (brain Endothelial Cell Line)-derived exosomes can pass through the blood–brain barrier and reduce VEGF levels *in vivo* by delivering drug to a brain tumor. This, in turn, result to a significant decrease in the tumor size (122). In a murine lung cancer model, cow milk exosomes were subjected for drug delivery for lung cancer. After injection of exosomes loaded with aferin-A, a tumor inhibitory effect was observed at doses lower in compare to unencapsulated drug (123).

The biological properties of exosomes give them with a valuable potential in medical research including cancer therapy. For example, since exosomes shuttle tumor-specific antigens, can be also attractive as anticancer vaccines (109). Given that uptake of TEXs is organotropism and performed through integrin-mediated signaling (8), thus blocking integrins through decoy peptides can be a good strategy to inhibit exosome fusion and uptake, subsequently result in blocking of tumor progression (124).

One of the recent immunotherapy method in treatment of lung cancer rely on blocking negative regulators of T-cell activation such as PD-1 and PD-L1 and inflammatory signals in the tumor microenvironment which can be mediated and reinforced by exosomes (110, 125). Another approaches such as blocking exosomal release or inhibition of the exosome-mediated cellular crosstalk in the tumor environment may be appropriate in suppress the development of a favorable tumor microenvironment (109). On the other hand, exosomes may modulate anti-inflammatory signals within the tumor microenvironment which may effectively enhance the efficacy of immunotherapy in lung cancer (126).

Overall, exosomes are starting to be considered in medical research especially in cancer diagnosis and treatment. Because of their unique biological properties, such as specific targeting, small size, shuttling signaling, and biological molecules, as well as the ability to cross biological barriers; exosomes can have a range of applications from diagnosis biomarkers to drug delivery and tumor immunotherapy. Despite some limitations in exosome usage, such as inconvenient nature of their isolation and purification methods, it is anticipated that exosomes will be utilized in cancer therapy in the near future. However, further more sophisticated clinical studies that address these current limitations in exosome biology is needed for translation of exosome-based technologies to clinical application.

## Conclusion

Exosomes mediate cross talk between the cells and their surrounding environment in normal and pathological conditions. TEXs are emerging as the major mechanism for communication between cancerous cells and the tumor microenvironment, which has a significant effect in tumor progression and metastasis. The data obtained to date using analytes within TEX as potential markers for the diagnosis and outcomes of lung cancer has provided much insight although further research is still required. The clinical use of TEX will open a new window to lung cancer management and treatment in the near future.

## Author Contributions

SA wrote first draft. EM and MV revised the manuscript. MM, AK, JG, and IA has revised final version and added extra information.

## Conflict of Interest Statement

The authors declare that the research was conducted in the absence of any commercial or financial relationships that could be construed as a potential conflict of interest.

## References

[1] Oser MG Niederst MJ Sequist LV Engelman JA. Transformation from non-small-cell lung cancer to small-cell lung cancer: molecular drivers and cells of origin. Lancet Oncol (2015) 16(4):e165–72. 10.1016/S1470-2045(14)71180-5 25846096 PMC4470698

[2] Bhatnagar S Schorey JS. Exosomes released from infected macrophages contain *Mycobacterium avium* glycopeptidolipids and are proinflammatory. J Biol Chem (2007) 282(35):25779–89. 10.1074/jbc.M702277200 17591775 PMC3636815

[3] Andre F Andersen M Wolfers J Lozier A Raposo G Serra V Exosomes in cancer immunotherapy: preclinical data. Adv Exp Med Biol (2001) 495:349–54. 10.1007/978-1-4615-0685-0_49 11774591

[4] Muller L Mitsuhashi M Simms P Gooding WE Whiteside TL. Tumor-derived exosomes regulate expression of immune function-related genes in human T cell subsets. Sci Rep (2016) 6:20254. 10.1038/srep20254 26842680 PMC4740743

[5] Sun T Kalionis B Lv G Xia S Gao W. Role of exosomal noncoding RNAs in lung carcinogenesis. Biomed Res Int (2015) 2015:125807. 10.1155/2015/125807 26583084 PMC4637011

[6] Zhang Y Wang X-F. A niche role for cancer exosomes in metastasis. Nat Cell Biol (2015) 17(6):709–11. 10.1038/ncb3181 26022917

[7] Sceneay J Smyth MJ Moller A. The pre-metastatic niche: finding common ground. Cancer Metastasis Rev (2013) 32(3–4):449–64. 10.1007/s10555-013-9420-1 23636348

[8] Hoshino A Costa-Silva B Shen TL Rodrigues G Hashimoto A Tesic Mark M Tumour exosome integrins determine organotropic metastasis. Nature (2015) 527(7578):329–35. 10.1038/nature15756 26524530 PMC4788391

[9] Théry C Ostrowski M Segura E. Membrane vesicles as conveyors of immune responses. Nat Rev Immunol (2009) 9(8):581–93. 10.1038/nri2567 19498381

[10] Viaud S Terme M Flament C Taieb J Andre F Novault S Dendritic cell-derived exosomes promote natural killer cell activation and proliferation: a role for NKG2D ligands and IL-15Ralpha. PLoS One (2009) 4(3):e4942. 10.1371/journal.pone.0004942 19319200 PMC2657211

[11] Chew V Toh HC Abastado JP. Immune microenvironment in tumor progression: characteristics and challenges for therapy. J Oncol (2012) 2012:608406. 10.1155/2012/608406 22927846 PMC3423944

[12] Pattabiraman DR Weinberg RA. Tackling the cancer stem cells – what challenges do they pose? Nat Rev Drug Discov (2014) 13(7):497–512. 10.1038/nrd4253 24981363 PMC4234172

[13] Haratani K Hayashi H Tanaka T Kaneda H Togashi Y Sakai K Tumor immune microenvironment and nivolumab efficacy in EGFR mutation-positive non-small cell lung cancer based on T790M status after disease progression during EGFR-TKI treatment. Ann oncol (2017) 28(7):1532–9. 10.1093/annonc/mdx183 28407039

[14] Whiteside TL Tumor-derived exosomes and their role in tumor-induced immune suppression. Vaccines (Basel) (2016) 4(4):35 10.3390/vaccines4040035 PMC5192355 27775593

[15] Kadota T Yoshioka Y Fujita Y Kuwano K Ochiya T. Extracellular vesicles in lung cancer-from bench to bedside. Semin Cell Dev Biol (2017) 67:39–47. 10.1016/j.semcdb.2017.03.001 28267596

[16] Fabbri M Paone A Calore F Galli R Gaudio E Santhanam R MicroRNAs bind to Toll-like receptors to induce prometastatic inflammatory response. Proc Natl Acad Sci U S A (2012) 109(31):E2110–6. 10.1073/pnas.1209414109 22753494 PMC3412003

[17] Malmberg K-J Carlsten M Björklund A Sohlberg E Bryceson YT Ljunggren H-G. Natural killer cell-mediated immunosurveillance of human cancer. Semin Immunol (2017) 31:20–9. 10.1016/j.smim.2017.08.002 28888619

[18] Berchem G Noman MZ Bosseler M Paggetti J Baconnais S Le Cam E Hypoxic tumor-derived microvesicles negatively regulate NK cell function by a mechanism involving TGF-β and miR23a transfer. Oncoimmunology (2015) 5(4):e1062968. 10.1080/2162402X.2015.1062968 27141372 PMC4839360

[19] Whiteside TL. Immune modulation of T-cell and NK (natural killer) cell activities by TEXs (tumour-derived exosomes). Biochem Soc Trans (2013) 41(1):245–51. 10.1042/BST20120265 23356291 PMC3721347

[20] Szczepanski MJ Szajnik M Welsh A Whiteside TL Boyiadzis M. Blast-derived microvesicles in sera from patients with acute myeloid leukemia suppress natural killer cell function via membrane-associated transforming growth factor-β1. Haematologica (2011) 96(9):1302–9. 10.3324/haematol.2010.039743 21606166 PMC3166100

[21] Cohnen A Chiang SC Stojanovic A Schmidt H Claus M Saftig P Surface CD107a/LAMP-1 protects natural killer cells from degranulation-associated damage. Blood (2013) 122(8):1411–8. 10.1182/blood-2012-07-441832 23847195

[22] Clayton A Mitchell JP Court J Linnane S Mason MD Tabi Z. Human tumor-derived exosomes down-modulate NKG2D expression. J Immunol (2008) 180(11):7249–58. 10.4049/jimmunol.180.11.7249 18490724

[23] Ashiru O Boutet P Fernández-Messina L Agüera-González S Skepper JN Valés-Gómez M Natural killer cell cytotoxicity is suppressed by exposure to the human NKG2D ligand MICA* 008 that is shed by tumor cells in exosomes. Cancer Res (2010) 70(2):481–9. 10.1158/0008-5472.CAN-09-1688 20068167 PMC2817492

[24] Liu Y Xiang X Zhuang X Zhang S Liu C Cheng Z Contribution of MyD88 to the tumor exosome-mediated induction of myeloid derived suppressor cells. Am J Pathol (2010) 176(5):2490–9. 10.2353/ajpath.2010.090777 20348242 PMC2861113

[25] Deng W Gowen BG Zhang L Wang L Lau S Iannello A A shed NKG2D ligand that promotes natural killer cell activation and tumor rejection. Science (2015) 348(6230):136–9. 10.1126/science.1258867 25745066 PMC4856222

[26] Filipazzi P Bürdek M Villa A Rivoltini L Huber V. Recent advances on the role of tumor exosomes in immunosuppression and disease progression. Semin Cancer Biol (2012) 22(4):342–9. 10.1016/j.semcancer.2012.02.005 22369922

[27] Valenti R Huber V Filipazzi P Iero M Parmiani G Rivoltini L Tumor-derived exosomes as dendritic cell modulators. In: Salter R Shurin M, editors. Dendritic Cells in Cancer. New York, NY: Springer (2009). p. 119–28. 10.1007/978-0-387-88611-4_8

[28] Huang S-H Li Y Zhang J Rong J Ye S. Epidermal growth factor receptor-containing exosomes induce tumor-specific regulatory T cells. Cancer Invest (2013) 31(5):330–5. 10.3109/07357907.2013.789905 23614656

[29] Zhou M Chen J Zhou L Chen W Ding G Cao L. Pancreatic cancer derived exosomes regulate the expression of TLR4 in dendritic cells via miR-203. Cell Immunol (2014) 292(1):65–9. 10.1016/j.cellimm.2014.09.004 25290620

[30] Yang C Kim S-H Bianco NR Robbins PD. Tumor-derived exosomes confer antigen-specific immunosuppression in a murine delayed-type hypersensitivity model. PLoS One (2011) 6(8):e22517. 10.1371/journal.pone.0022517 21829629 PMC3149056

[31] Chen W Jiang J Xia W Huang J. Tumor-related exosomes contribute to tumor-promoting microenvironment: an immunological perspective. J Immunol Res (2017) 2017:1073947. 10.1155/2017/1073947 28642882 PMC5470026

[32] Gabrilovich DI Nagaraj S. Myeloid-derived-suppressor cells as regulators of the immune system. Nat Rev Immunol (2009) 9(3):162. 10.1038/nri2506 19197294 PMC2828349

[33] Liu Y Gu Y Cao X. The exosomes in tumor immunity. Oncoimmunology (2015) 4(9):e1027472. 10.1080/2162402X.2015.1027472 26405598 PMC4570093

[34] Valenti R Huber V Filipazzi P Pilla L Sovena G Villa A Human tumor-released microvesicles promote the differentiation of myeloid cells with transforming growth factor-β-mediated suppressive activity on T lymphocytes. Cancer Res (2006) 66(18):9290–8. 10.1158/0008-5472.CAN-06-1819 16982774

[35] Valenti R Huber V Iero M Filipazzi P Parmiani G Rivoltini L. Tumor-released microvesicles as vehicles of immunosuppression. Cancer Res (2007) 67(7):2912–5. 10.1158/0008-5472.CAN-07-0520 17409393

[36] Xiang X Poliakov A Liu C Liu Y Deng ZB Wang J Induction of myeloid-derived suppressor cells by tumor exosomes. Int J Cancer (2009) 124(11):2621–33. 10.1002/ijc.24249 19235923 PMC2757307

[37] Chalmin F Ladoire S Mignot G Vincent J Bruchard M Remy-Martin JP Membrane-associated Hsp72 from tumor-derived exosomes mediates STAT3-dependent immunosuppressive function of mouse and human myeloid-derived suppressor cells. J Clin Invest (2010) 120(2):457–71. 10.1172/JCI40483 20093776 PMC2810085

[38] Mignot G Chalmin F Ladoire S Rébé C Ghiringhelli F Xiang X Tumor exosome-mediated MDSC activation. Am J Pathol (2011) 178(3):1403–5. 10.1016/j.ajpath.2010.11.078 21356390 PMC3069877

[39] Xiang X Liu Y Zhuang X Zhang S Michalek S Taylor DD TLR2-mediated expansion of MDSCs is dependent on the source of tumor exosomes. Am J Pathol (2010) 177(4):1606–10. 10.2353/ajpath.2010.100245 20802178 PMC2947257

[40] Chanmee T Ontong P Konno K Itano N. Tumor-associated macrophages as major players in the tumor microenvironment. Cancers (2014) 6(3):1670–90. 10.3390/cancers6031670 25125485 PMC4190561

[41] Yang L Zhang Y. Tumor-associated macrophages: from basic research to clinical application. J Hematol Oncol (2017) 10(1):58. 10.1186/s13045-017-0430-2 28241846 PMC5329931

[42] Song X Ding Y Liu G Yang X Zhao R Zhang Y Cancer cell-derived exosomes induce mitogen-activated protein kinase-dependent monocyte survival by transport of functional receptor tyrosine kinases. J Biol Chem (2016) 291(16):8453–64. 10.1074/jbc.M116.716316 26895960 PMC4861419

[43] Chow A Zhou W Liu L Fong MY Champer J Van Haute D Macrophage immunomodulation by breast cancer-derived exosomes requires Toll-like receptor 2-mediated activation of NF-κB. Sci Rep (2014) 4:5750 10.1038/srep05750 25034888 PMC4102923

[44] Menck K Klemm F Gross JC Pukrop T Wenzel D Binder C. Induction and transport of Wnt 5a during macrophage-induced malignant invasion is mediated by two types of extracellular vesicles. Oncotarget (2013) 4(11):2057. 10.18632/oncotarget.1336 24185202 PMC3875769

[45] Ades PA A controversial step forward: a commentary on the 2013 ACC/AHA guideline on the treatment of blood cholesterol to reduce atherosclerotic cardiovascular risk in adults. Coron Artery Dis (2014) 25(4):360–3. 10.1097/MCA.0000000000000086 24518291 PMC4107357

[46] Iriki T Ohnishi K Fujiwara Y Horlad H Saito Y Pan C The cell-cell interaction between tumor-associated macrophages and small cell lung cancer cells is involved in tumor progression via STAT3 activation. Lung Cancer (2017) 106:22–32. 10.1016/j.lungcan.2017.01.003 28285690

[47] Zhu Y Chen X Pan Q Wang Y Su S Jiang C A comprehensive proteomics analysis reveals a secretory path-and status-dependent signature of exosomes released from tumor-associated macrophages. J Proteome Res (2015) 14(10):4319–31. 10.1021/acs.jproteome.5b00770 26312558

[48] Wu Q Wu X Ying X Zhu Q Wang X Jiang L Suppression of endothelial cell migration by tumor associated macrophage-derived exosomes is reversed by epithelial ovarian cancer exosomal lncRNA. Cancer Cell Int (2017) 17:62. 10.1186/s12935-017-0430-x 28592924 PMC5461704

[49] Zheng P Chen L Yuan X Luo Q Liu Y Xie G Exosomal transfer of tumor-associated macrophage-derived miR-21 confers cisplatin resistance in gastric cancer cells. J Exp Clin Cancer Res (2017) 36(1):53. 10.1186/s13046-017-0528-y 28407783 PMC5390430

[50] Muller L Simms P Hong C-S Nishimura MI Jackson EK Watkins SC Human tumor-derived exosomes (TEX) regulate Treg functions via cell surface signaling rather than uptake mechanisms. Oncoimmunology (2017) 6(8):e1261243. 10.1080/2162402X.2016.1261243 28919985 PMC5593709

[51] Brimnes MK Vangsted AJ Knudsen LM Gimsing P Gang AO Johnsen HE Increased level of both CD4+ FOXP3+ regulatory T cells and CD14+ HLA-DR−/low myeloid-derived suppressor cells and decreased level of dendritic cells in patients with multiple myeloma. Scand J Immunol (2010) 72(6):540–7. 10.1111/j.1365-3083.2010.02463.x 21044128

[52] Szajnik M Czystowska M Szczepanski MJ Mandapathil M Whiteside TL. Tumor-derived microvesicles induce, expand and up-regulate biological activities of human regulatory T cells (Treg). PLoS One (2010) 5(7):e1146. 10.1371/journal.pone.0011469 20661468 PMC2908536

[53] Wieckowski EU Visus C Szajnik M Szczepanski MJ Storkus WJ Whiteside TL. Tumor-derived microvesicles promote regulatory T cell expansion and induce apoptosis in tumor-reactive activated CD8+ T lymphocytes. J Immunol (2009) 183(6):3720–30. 10.4049/jimmunol.0900970 19692638 PMC3721354

[54] Clayton A Al-Taei S Webber J Mason MD Tabi Z. Cancer exosomes express CD39 and CD73, which suppress T cells through adenosine production. J Immunol (2011) 187(2):676–83. 10.4049/jimmunol.1003884 21677139

[55] Rosser EC Mauri C. Regulatory B cells: origin, phenotype, and function. Immunity (2015) 42(4):607–12. 10.1016/j.immuni.2015.04.005 25902480

[56] Guan H Wan Y Lan J Wang Q Wang Z Li Y PD-L1 is a critical mediator of regulatory B cells and T cells in invasive breast cancer. Sci Rep (2016) 6:35651. 10.1038/srep35651 27762298 PMC5071845

[57] Yang C Chalasani G Ng Y-H Robbins PD. Exosomes released from *Mycoplasma* infected tumor cells activate inhibitory B cells. PLoS One (2012) 7(4):e36138. 10.1371/journal.pone.0036138 22558358 PMC3338602

[58] Li Y An J Huang S He J Zhang J. Esophageal cancer-derived microvesicles induce regulatory B cells. Cell Biochem Funct (2015) 33(5):308–13. 10.1002/cbf.3115 26009869

[59] Lamouille S Xu J Derynck R Molecular mechanisms of epithelial–mesenchymal transition. Nat Rev Mol Cell Biol (2014) 15(3):178 10.1038/nrm3758 24556840 PMC4240281

[60] Xiao D He J. Epithelial mesenchymal transition and lung cancer. J Thorac Dis (2010) 2(3):154–9. 10.3978/j.issn.2072-1439.2010.02.03.7 22263037 PMC3256459

[61] Ni M Shi X-L Qu Z-G Jiang H Chen Z-Q Hu J. Epithelial mesenchymal transition of non–small–cell lung cancer cells A549 induced by SPHK1. Asian Pac J Trop Med (2015) 8(2):142–6. 10.1016/S1995-7645(14)60305-9 25902029

[62] Lee JM Dedhar S Kalluri R Thompson EW. The epithelial–mesenchymal transition: new insights in signaling, development, and disease. J Cell Biol (2006) 172(7):973–81. 10.1083/jcb.200601018 16567498 PMC2063755

[63] Rahman MA Barger JF Lovat F Gao M Otterson GA Nana-Sinkam P. Lung cancer exosomes as drivers of epithelial mesenchymal transition. Oncotarget (2016) 7(34):54852. 10.18632/oncotarget.10243 27363026 PMC5342386

[64] Satelli A Li S. Vimentin in cancer and its potential as a molecular target for cancer therapy. Cell Mol Life Sci (2011) 68(18):3033–46. 10.1007/s00018-011-0735-1 21637948 PMC3162105

[65] Havel LS Kline ER Salgueiro AM Marcus AI. Vimentin regulates lung cancer cell adhesion through a VAV2–Rac1 pathway to control focal adhesion kinase activity. Oncogene (2015) 34(15):1979–90. 10.1038/onc.2014.123 24858039 PMC4245398

[66] Tadokoro A Kanaji N Liu D Yokomise H Haba R Ishii T Vimentin regulates invasiveness and is a poor prognostic marker in non-small cell lung cancer. Anticancer Res (2016) 36(4):1545–51. 27069130

[67] Wei J Xu G Wu M Zhang Y Li Q Liu P Overexpression of vimentin contributes to prostate cancer invasion and metastasis via src regulation. Anticancer Res (2008) 28(1A):327–34. 18383865

[68] Zhao Y Yan Q Long X Chen X Wang Y. Vimentin affects the mobility and invasiveness of prostate cancer cells. Cell Biochem Funct (2008) 26(5):571–7. 10.1002/cbf.1478 18464297

[69] Otsuki S Inokuchi M Enjoji M Ishikawa T Takagi Y Kato K Vimentin expression is associated with decreased survival in gastric cancer. Oncol Rep (2011) 25(5):1235–42. 10.3892/or.2011.1185 21327330

[70] Kim J Kim TY Lee MS Mun JY Ihm C Kim SA. Exosome cargo reflects TGF-β1-mediated epithelial-to-mesenchymal transition (EMT) status in A549 human lung adenocarcinoma cells. Biochem Biophys Res Commun (2016) 478(2):643–8. 10.1016/j.bbrc.2016.07.124 27492069

[71] Cao M Seike M Soeno C Mizutani H Kitamura K Minegishi Y MiR-23a regulates TGF-β-induced epithelial-mesenchymal transition by targeting E-cadherin in lung cancer cells. Int J Oncol (2012) 41(3):869–75. 10.3892/ijo.2012.1535 22752005 PMC3582905

[72] Felicetti F Feo A Coscia C Puglisi R Pedini F Pasquini L Exosome-mediated transfer of miR-222 is sufficient to increase tumor malignancy in melanoma. J Transl Med (2016) 14(1):56. 10.1186/s12967-016-0811-2 26912358 PMC4765208

[73] Cui H Seubert B Stahl E Dietz H Reuning U Moreno-Leon L Tissue inhibitor of metalloproteinases-1 induces a pro-tumourigenic increase of miR-210 in lung adenocarcinoma cells and their exosomes. Oncogene (2015) 34(28):3640–50. 10.1038/onc.2014.300 25263437

[74] Valadi H Ekström K Bossios A Sjöstrand M Lee JJ Lötvall JO. Exosome-mediated transfer of mRNAs and microRNAs is a novel mechanism of genetic exchange between cells. Nat Cell Biol (2007) 9(6):654–9. 10.1038/ncb1596 17486113

[75] Hsu YL Hung JY Chang WA Lin YS Pan YC Tsai PH Hypoxic lung cancer-secreted exosomal miR-23a increased angiogenesis and vascular permeability by targeting prolyl hydroxylase and tight junction protein ZO-1. Oncogene (2017) 36(34):4929–42. 10.1038/onc.2017.105 28436951

[76] Chang Y-H Chiu Y-J Cheng H-C Liu F-J Lai W-W Chang H-J Down-regulation of TIMP-1 inhibits cell migration, invasion, and metastatic colonization in lung adenocarcinoma. Tumor Biol (2015) 36(5):3957–67. 10.1007/s13277-015-3039-5 25578494

[77] Iniesta P Morán A De Juan C Gómez A Hernando F García-Aranda C Biological and clinical significance of MMP-2, MMP-9, TIMP-1 and TIMP-2 in non-small cell lung cancer. Oncol Rep (2007) 17(1):217–23. 10.3892/or.17.1.217 17143501

[78] Pesta M Kulda V Kucera R Pesek M Vrzalova J Liska V Prognostic significance of TIMP-1 in non-small cell lung cancer. Anticancer Res (2011) 31(11):4031–8. 22110238

[79] Safranek J Pesta M Holubec L Kulda V Dreslerova J Vrzalova J Expression of MMP-7, MMP-9, TIMP-1 and TIMP-2 mRNA in lung tissue of patients with non-small cell lung cancer (NSCLC) and benign pulmonary disease. Anticancer Res (2009) 29(7):2513–7. 19596921

[80] Rabinowits G Gerçel-Taylor C Day JM Taylor DD Kloecker GH. Exosomal microRNA: a diagnostic marker for lung cancer. Clin Lung Cancer (2009) 10(1):42–6. 10.3816/CLC.2009.n.006 19289371

[81] Yanaihara N Caplen N Bowman E Seike M Kumamoto K Yi M Unique microRNA molecular profiles in lung cancer diagnosis and prognosis. Cancer Cell (2006) 9(3):189–98. 10.1016/j.ccr.2006.01.025 16530703

[82] Al-Nedawi K Meehan B Kerbel RS Allison AC Rak J. Endothelial expression of autocrine VEGF upon the uptake of tumor-derived microvesicles containing oncogenic EGFR. Proc Natl Acad Sci U S A (2009) 106(10):3794–9. 10.1073/pnas.0804543106 19234131 PMC2656159

[83] Sheu JJ Lee FY Wallace CG Tsai TH Leu S Chen YL Administered circulating microparticles derived from lung cancer patients markedly improved angiogenesis, blood flow and ischemic recovery in rat critical limb ischemia. J Transl Med (2015) 13:59. 10.1186/s12967-015-0381-8 25889721 PMC4369091

[84] Liu Y Cao X. Characteristics and significance of the pre-metastatic niche. Cancer Cell (2016) 30(5):668–81. 10.1016/j.ccell.2016.09.011 27846389

[85] Antonopoulos D Balatsos NAA Gourgoulianis KI Cancer’s smart bombs: tumor-derived exosomes target lung epithelial cells triggering pre-metastatic niche formation. J Thorac Dis (2017) 9(4):969–72. 10.21037/jtd.2017.03.129 28523150 PMC5418243

[86] Kaplan RN Rafii S Lyden D Preparing the “soil”: the premetastatic niche. Cancer Res (2006) 66(23):11089–93. 10.1158/0008-5472.CAN-06-2407 17145848 PMC2952469

[87] Huang Y Song N Ding Y Yuan S Li X Cai H Pulmonary vascular destabilization in the premetastatic phase facilitates lung metastasis. Cancer Res (2009) 69(19):7529–37. 10.1158/0008-5472.CAN-08-4382 19773447

[88] Peinado H Zhang H Matei IR Costa-Silva B Hoshino A Rodrigues G Pre-metastatic niches: organ-specific homes for metastases. Nat Rev Cancer (2017) 17(5):302–17. 10.1038/nrc.2017.6 28303905

[89] Lobb RJ Lima LG Möller A. Exosomes: key mediators of metastasis and pre-metastatic niche formation. Semin Cell Dev Biol (2017) 67:3–10. 10.1016/j.semcdb.2017.01.004 28077297

[90] Weidle UH Birzele F Kollmorgen G Rueger R The multiple roles of exosomes in metastasis. Cancer Genomics Proteomics (2017) 14(1):1–15. 10.21873/cgp.20015 28031234 PMC5267497

[91] Janowska-Wieczorek A Wysoczynski M Kijowski J Marquez-Curtis L Machalinski B Ratajczak J Microvesicles derived from activated platelets induce metastasis and angiogenesis in lung cancer. Int J Cancer (2005) 113(5):752–60. 10.1002/ijc.20657 15499615

[92] Grange C Tapparo M Collino F Vitillo L Damasco C Deregibus MC Microvesicles released from human renal cancer stem cells stimulate angiogenesis and formation of lung premetastatic niche. Cancer Res (2011) 71(15):5346–56. 10.1158/0008-5472.CAN-11-0241 21670082

[93] Liu Y Gu Y Han Y Zhang Q Jiang Z Zhang X Tumor exosomal RNAs promote lung pre-metastatic niche formation by activating alveolar epithelial TLR3 to recruit neutrophils. Cancer Cell (2016) 30(2):243–56. 10.1016/j.ccell.2016.06.021 27505671

[94] Hiratsuka S Watanabe A Sakurai Y Akashi-Takamura S Ishibashi S Miyake K The S100A8-serum amyloid A3-TLR4 paracrine cascade establishes a pre-metastatic phase. Nat Cell Biol (2008) 10(11):1349–55. 10.1038/ncb1794 18820689

[95] Peinado H Aleckovic M Lavotshkin S Matei I Costa-Silva B Moreno-Bueno G Melanoma exosomes educate bone marrow progenitor cells toward a pro-metastatic phenotype through MET. Nat Med (2012) 18(6):883–91. 10.1038/nm.2753 22635005 PMC3645291

[96] Taverna S Pucci M Giallombardo M Di Bella MA Santarpia M Reclusa P Amphiregulin contained in NSCLC-exosomes induces osteoclast differentiation through the activation of EGFR pathway. Sci Rep (2017) 7(1):3170. 10.1038/s41598-017-03460-y 28600504 PMC5466625

[97] Dempke WC Suto T Reck M. Targeted therapies for non-small cell lung cancer. Lung Cancer (2010) 67(3):257–74. 10.1016/j.lungcan.2009.10.012 19914732

[98] Higginbotham JN Beckler MD Gephart JD Franklin JL Bogatcheva G Kremers G-J Amphiregulin exosomes increase cancer cell invasion. Curr Biol (2011) 21(9):779–86. 10.1016/j.cub.2011.03.043 21514161 PMC3417320

[99] Furugaki K Moriya Y Iwai T Yorozu K Yanagisawa M Kondoh K Erlotinib inhibits osteolytic bone invasion of human non-small-cell lung cancer cell line NCI-H292. Clin Exp Metastasis (2011) 28(7):649–59. 10.1007/s10585-011-9398-4 21688034 PMC3198194

[100] Nogués L Benito-Martin A Hergueta-Redondo M Peinado H. The influence of tumour-derived extracellular vesicles on local and distal metastatic dissemination. Mol Aspects Med (2018) 60:15–26. 10.1016/j.mam.2017.11.012 29196097 PMC5856602

[101] Macklin R Wang H Loo D Martin S Cumming A Cai N Extracellular vesicles secreted by highly metastatic clonal variants of osteosarcoma preferentially localize to the lungs and induce metastatic behaviour in poorly metastatic clones. Oncotarget (2016) 7(28):43570. 10.18632/oncotarget.9781 27259278 PMC5190045

[102] Jin X Mu P. Targeting breast cancer metastasis. Breast Cancer (Auckl) (2015) 9(Suppl 1):23–34. 10.4137/BCBCR.S25460 26380552 PMC4559199

[103] Jin Y Ai J Shi J. Lung microenvironment promotes the metastasis of human hepatocellular carcinoma cells to the lungs. Int J Clin Exp Med (2015) 8(6):9911. 26309675 PMC4538034

[104] Macklin R Wang H Loo D Martin S Cumming A Cai N Extracellular vesicles secreted by highly metastatic clonal variants of osteosarcoma preferentially localize to the lungs and induce metastatic behaviour in poorly metastatic clones. Oncotarget (2016) 7(28):43570–87. 10.18632/oncotarget.9781 27259278 PMC5190045

[105] Alberg AJ Samet JM Epidemiology of lung cancer. Chest (2003) 123(1):21S–49S. 10.1378/chest.123.1_suppl.21S 12527563

[106] Soung YH Ford S Zhang V Chung J Exosomes in cancer diagnostics. Cancers (2017) 9(1):8 10.3390/cancers9010008 PMC5295779 28085080

[107] Alipoor SD Mortaz E Garssen J Movassaghi M Mirsaeidi M Adcock IM. Exosomes and exosomal miRNA in respiratory diseases. Mediators Inflamm (2016) 2016:5628404. 10.1155/2016/5628404 27738390 PMC5055958

[108] Zöller M Exosomes in cancer disease. Methods Mol Biol (2016) 1381:111–49. 10.1007/978-1-4939-3204-7_7 26667458

[109] Inamdar S Nitiyanandan R Rege K. Emerging applications of exosomes in cancer therapeutics and diagnostics. Bioeng Transl Med (2017) 2(1):70–80. 10.1002/btm2.10059 28529978 PMC5413841

[110] Sandfeld-Paulsen B Aggerholm-Pedersen N Baek R Jakobsen K Meldgaard P Folkersen B Exosomal proteins as prognostic biomarkers in non-small cell lung cancer. Mol Oncol (2016) 10(10):1595–602. 10.1016/j.molonc.2016.10.003 27856179 PMC5423137

[111] Dejima H Iinuma H Kanaoka R Matsutani N Kawamura M Exosomal microRNA in plasma as a non-invasive biomarker for the recurrence of non-small cell lung cancer. Oncol Lett (2017) 13(3):1256–63. 10.3892/ol.2017.5569 28454243 PMC5403401

[112] Brinkmann K Enderle D Koestler T Bentink S Emenegger J Spiel A Plasma-based diagnostics for detection of EML4-ALK fusion transcripts in NSCLC patients. AACR (2015) 75(15 Suppl):Abstract nr 545.

[113] Reclusa P Sirera R Araujo A Giallombardo M Valentino A Sorber L Exosomes genetic cargo in lung cancer: a truly Pandora’s box. Transl Lung Cancer Res (2016) 5(5):483–91. 10.21037/tlcr.2016.10.06 27826529 PMC5099517

[114] Alipoor SD Adcock IM Garssen J Mortaz E Varahram M Mirsaeidi M The roles of miRNAs as potential biomarkers in lung diseases. Eur J Pharmacol (2016) 791:395–404. 10.1016/j.ejphar.2016.09.015 27634639 PMC7094636

[115] Cazzoli R Buttitta F Di Nicola M Malatesta S Marchetti A Rom WN MicroRNAs derived from circulating exosomes as noninvasive biomarkers for screening and diagnosing lung cancer. J Thorac Oncol (2013) 8(9):1156–62. 10.1097/JTO.0b013e318299ac32 23945385 PMC4123222

[116] Munagala R Aqil F Gupta RC. Exosomal miRNAs as biomarkers of recurrent lung cancer. Tumor Biol (2016) 37(8):10703–14. 10.1007/s13277-016-4939-8 26867772

[117] Park J Hwang M Choi B Jeong H Jung JH Kim HK Exosome classification by pattern analysis of surface-enhanced Raman spectroscopy data for lung cancer diagnosis. Anal Chem (2017) 89(12):6695–701. 10.1021/acs.analchem.7b00911 28541032

[118] Ueda K Ishikawa N Tatsuguchi A Saichi N Fujii R Nakagawa H. Antibody-coupled monolithic silica microtips for high throughput molecular profiling of circulating exosomes. Sci Rep (2014) 4:6232. 10.1038/srep06232 25167841 PMC4148700

[119] Jakobsen KR Paulsen BS Baek R Varming K Sorensen BS Jørgensen MM. Exosomal proteins as potential diagnostic markers in advanced non-small cell lung carcinoma. J Extracell Vesicles (2015) 4(1):26659. 10.3402/jev.v4.26659 25735706 PMC4348413

[120] Li Y Zhang Y Qiu F Qiu Z. Proteomic identification of exosomal LRG1: a potential urinary biomarker for detecting NSCLC. Electrophoresis (2011) 32(15):1976–83. 10.1002/elps.201000598 21557262

[121] Sandfeld-Paulsen B Jakobsen KR Baek R Folkersen BH Rasmussen TR Meldgaard P Exosomal proteins as diagnostic biomarkers in lung cancer. J Thorac Oncol (2016) 11(10):1701–10. 10.1016/j.jtho.2016.05.034 27343445

[122] Yang T Martin P Fogarty B Brown A Schurman K Phipps R Exosome delivered anticancer drugs across the blood-brain barrier for brain cancer therapy in *Danio rerio*. Pharm Res (2015) 32(6):2003–14. 10.1007/s11095-014-1593-y 25609010 PMC4520542

[123] Munagala R Aqil F Jeyabalan J Gupta RC. Bovine milk-derived exosomes for drug delivery. Cancer Lett (2016) 371(1):48–61. 10.1016/j.canlet.2015.10.020 26604130 PMC4706492

[124] Wu YJ Muldoon LL Gahramanov S Kraemer DF Marshall DJ Neuwelt EA Targeting alphaV-integrins decreased metastasis and increased survival in a nude rat breast cancer brain metastasis model. J Neurooncol (2012) 110(1):27–36. 10.1007/s11060-012-0942-0 22842979 PMC3726254

[125] Zhang H-G Grizzle WE. Exosomes and cancer: a newly described pathway of immune suppression. Clin Cancer Res (2011) 17(5):959–64. 10.1158/1078-0432.CCR-10-1489 21224375 PMC3155407

[126] Whiteside TL. Tumor-derived exosomes and their role in cancer progression. Adv Clin Chem (2016) 74:103–41. 10.1016/bs.acc.2015.12.005 27117662 PMC5382933

